# Children With Medulloblastoma: Surgical Outcomes and Survival Challenges

**DOI:** 10.7759/cureus.109643

**Published:** 2026-05-25

**Authors:** Armando Francisco Perez Castell, Jorge de la Salud Rodríguez Rodríguez, Elizabeth Meza Mata, Enrique López Facio, Fany Karina Segura López

**Affiliations:** 1 Neurosurgery and Spine Surgery, Star Médica/Universidad Autónoma de Chihuahua, Chihuahua, MEX; 2 Neurosurgery, High Specialty Medical Unit No. 71, Mexican Social Security Institute (IMSS), Torreón, MEX; 3 Anatomical Pathology, High Specialty Medical Unit No. 71, Mexican Social Security Institute (IMSS), Torreón, MEX; 4 Oncology, High Specialty Medical Unit No. 71, Mexican Social Security Institute (IMSS), Torreón, MEX; 5 Anesthesiology, High Specialty Medical Unit No. 71, Mexican Social Security Institute (IMSS), Torreón, MEX

**Keywords:** overall survival (os), pediatric medulloblastoma, retrospective cohort, risk factors, ‘surgical resection’

## Abstract

Background

Medulloblastoma is one of the most common malignant pediatric brain tumors and remains associated with significant morbidity and mortality despite multimodal treatment strategies.

Objective

This study aimed to evaluate the clinical characteristics and prognostic factors influencing overall survival in pediatric patients diagnosed with medulloblastoma.

Methods

This retrospective cohort study analyzed pediatric patients diagnosed with medulloblastoma who underwent surgical resection between 2018 and 2022. Demographic, radiologic, histopathologic, and therapeutic variables were collected. OS and associated prognostic factors were assessed using the Kaplan-Meier method.

Results

A total of 24 patients were included, with a mean age of 7.83 years; 66.7% were male. Headache was the most frequent presenting symptom (41.7%). Tumors were most commonly located in the cerebellar vermis (33.3%), and the desmoplastic variant was the predominant histological subtype (54%). Homogeneous contrast enhancement was observed in 62% of cases. Subtotal resection was performed in 79.2% of patients. Three patients were lost to follow-up. Among the remaining 21 patients, 13 died during the study period. Median overall survival was 32.28 months, with a 20-month overall survival rate of 57.2%. Total resection was associated with improved survival outcomes. Tumor location and histological subtype also influenced prognosis.

Conclusion

Medulloblastoma continues to demonstrate variable survival outcomes depending on tumor- and treatment-related factors. Surgical resection combined with adjuvant therapy remains essential in the management of pediatric patients with medulloblastoma.

## Introduction

Medulloblastoma is an embryonal tumor of the CNS that arises in the posterior fossa and predominantly affects the pediatric population [1]. It accounts for approximately 20% of all pediatric brain tumors and 63% of intracranial embryonal tumors in children aged 0 to 14 years [2], with an incidence rate of 0.5 cases per 100,000 children, or 5 cases per million pediatric patients [3]. In the United States, approximately 338 new pediatric cases are diagnosed annually. Juraschka K and Taylor MD reported a 5-year overall survival (OS) rate of 70% and a recurrence-free survival rate of 67% [4]. Dressler EV et al. reported age-stratified 5-year survival rates as follows: 47.4% for patients younger than 1 year, 64.5% for those aged 1 to 4 years, 74.2% for those aged 5 to 9 years, and 81.5% for those aged 10 to 19 years [5].

The etiology of medulloblastoma remains largely unknown, with most cases occurring sporadically. Potential contributing factors include environmental exposures, medications that interfere with embryonic development, and viral infections; however, definitive evidence is currently lacking [6]. Approximately 5% of pediatric cases are associated with genetic syndromes such as Gorlin syndrome, Turcot syndrome, and Li-Fraumeni syndrome. These tumors originate from primitive neuroectodermal cells involved in the development of the cerebellum and brainstem, most commonly arising in the external granular layer (EGL) of the cerebellum [3].

Histologically, medulloblastomas are classified into four subtypes: classic, desmoplastic/nodular (DNMB), medulloblastoma with extensive nodularity (MBEN), and large cell/anaplastic (LCA). The classic subtype accounts for more than 70% of cases [7]. At the molecular level, medulloblastomas are classified by the WHO into four subgroups: WNT-activated, SHH-activated TP53-wildtype, SHH-activated TP53-mutant, and non-WNT/non-SHH Groups 3 and 4. The most common subtypes in children are Group 4 (35-40%), SHH-activated (30%), Group 3 (20-25%), and WNT-activated (10%) [8]. While the genetic pathways underlying the WNT and SHH subgroups are well characterized, the pathobiology of Groups 3 and 4 remains under investigation [9].

Early surgical outcomes for medulloblastoma were poor. In his series of 61 cases, Cushing reported an operative mortality rate of 32% [10]. Surgical outcomes have since improved significantly, with contemporary series reporting mortality rates below 10% [11]. Unfortunately, treatment-related morbidity and mortality remain substantial, and even children who achieve remission or cure often experience long-term sequelae, including neurological, cognitive, and endocrinological impairments [12]. This study aims to contribute to the standardization and comparative analysis of existing global literature regarding the relationships among clinical, imaging, and histopathological data, which may aid in distinguishing between high-risk and low-risk cases. The goal is to provide the multidisciplinary medical team with actionable insights for improvement, thereby positively influencing the clinical course and survival outcomes of patients diagnosed with medulloblastoma at a tertiary care center.

## Materials and methods

Study design

A single-center retrospective cohort study was conducted using the clinical records of pediatric patients diagnosed with medulloblastoma who underwent surgical resection between 2018 and 2022 at a tertiary care center. Patients were identified through the pathology service database, and retrospective data were obtained from electronic medical records and pathology reports. Demographic data, clinical presentation, imaging findings, surgical approach, and OS were collected and analyzed. This study was approved by the institutional ethics committee.

Eligibility criteria and baseline characteristics

Due to the retrospective nature of the study and the use of anonymized clinical records, the requirement for informed consent was waived by the institutional ethics committee in accordance with the institutional ethical standards applicable to retrospective studies based exclusively on previously recorded clinical data. All patient information was anonymized and handled confidentially to protect the identity and privacy of each participant. Clinical data from patients younger than 18 years of age who were diagnosed with and treated for medulloblastoma between January 2018 and December 2022 at the study center were included. In addition, written informed consent for all diagnostic and therapeutic procedures, including biological sample collection, had been obtained from the parents or legal guardians of each patient before their performance. Patients were excluded if they had undergone surgery at another institution, lacked histopathological confirmation of medulloblastoma, or had incomplete clinical records or follow-up data.

Follow-up

All variables were obtained through a review of the electronic medical records of the included patients. Data collected included demographic characteristics, such as age and sex; clinical presentation, including symptoms; histological subtype; tumor location; contrast enhancement patterns on MRI; extent of surgical resection; and administration of chemotherapy and radiotherapy. Clinical records reviewed included brain MRI scans, preoperative and postoperative neuroaxis evaluations, and documentation related to adjuvant therapies. Assessment for disseminated disease was conducted at the time of diagnosis. The extent of resection was determined using contrast-enhanced postoperative MRI performed within 72 hours after surgery. Residual tumor burden was assessed according to the largest linear dimension of the residual enhancing lesion identified on postoperative imaging. Gross total resection and subtotal resection (STR) classifications were established using a residual tumor cut-off of 1.5 cm². Adjuvant chemotherapy and radiotherapy were administered according to multidisciplinary institutional evaluation and contemporary pediatric neuro-oncology treatment protocols, considering tumor characteristics and the patient’s clinical status. Follow-up information was obtained from neurosurgical and oncological outpatient records and imaging evaluations documented during routine follow-up care.

To minimize potential sources of bias, standardized data collection procedures were implemented across all cases. Data extraction was performed independently by two researchers, and discrepancies were resolved through consensus or consultation with a third reviewer. Selection bias was addressed by consistently applying predefined inclusion and exclusion criteria to all eligible patients. Information bias was reduced by relying exclusively on systematically reviewed electronic medical records, imaging studies, and pathology reports, using a structured data extraction form.

The study size was determined based on the total number of eligible patients diagnosed with medulloblastoma at the study center between January 2018 and December 2022. No formal sample size calculation was performed, as all available cases that met the inclusion criteria during the study period were included, thereby maximizing the representativeness and completeness of the cohort. Patients lost to follow-up were defined as those who discontinued outpatient follow-up evaluations and for whom no additional clinical information or subsequent medical records were available in the institutional files.

Statistical analysis

Data were recorded using a standardized data collection instrument and subsequently processed and analyzed using Microsoft Excel 2016. Missing data were addressed by conducting a complete-case analysis, including only patients with available information for all variables of interest. Variables with missing data were identified and reported, and their potential impact on the results was assessed. No imputation methods were applied due to the retrospective nature of the study and the relatively low proportion of missing data (<5% for all key variables).

Quantitative variables, such as age at diagnosis and tumor size, were analyzed as continuous variables. Tumor size was maintained as a continuous variable to preserve statistical power, except when analyzing associations with dissemination risk, where it was dichotomized at the mean value. Categorical variables, including clinical symptoms, sex, and tumor location, were analyzed using percentages to describe their distribution within the cohort. The Shapiro-Wilk test was applied to assess normality. Based on the data distribution, continuous variables were reported as mean ± standard deviation or as median and interquartile range.

OS was estimated using the Kaplan-Meier method, considering the time interval between the date of surgery and the date of death or last follow-up. Due to the limited sample size, only univariable survival analyses were performed using the Kaplan-Meier method and log-rank testing, with a significance level set at 5%. Subgroup analyses were conducted to assess survival outcomes according to histological subtype, tumor location, and extent of resection. A p-value <0.05 was considered statistically significant. All statistical analyses were performed using SPSS version 25.0.

## Results

Tumor localization and histological variants

**Figure 1:**
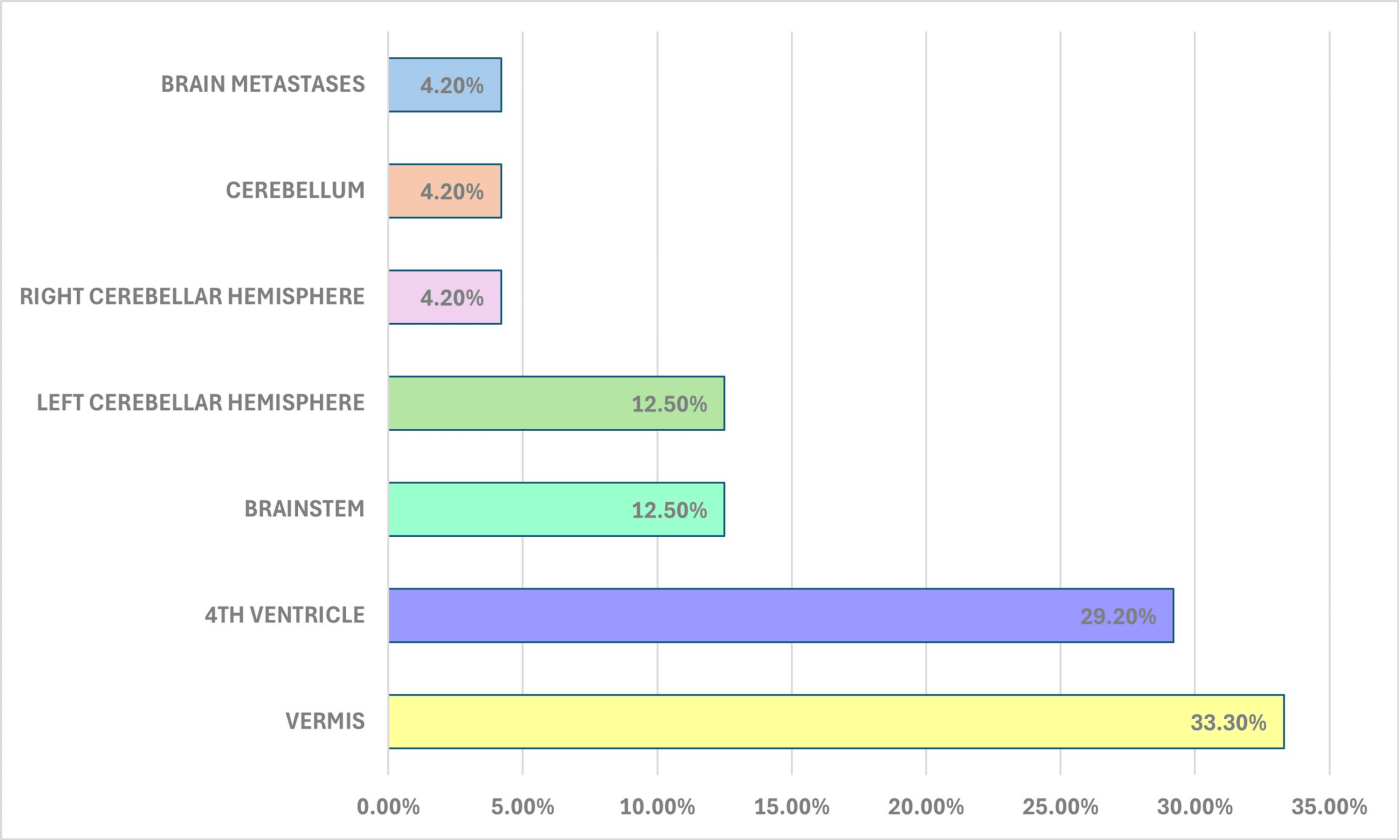
Medulloblastoma location. Anatomical distribution of primary tumor locations in pediatric medulloblastoma cases. The most frequent site was the cerebellar vermis (33.3%), followed by the fourth ventricle (29.2%), and the brainstem and left cerebellar hemisphere (12.5% each). Less common sites included the cerebellum, brain metastases, and right cerebellar hemisphere (4.2% each). Data are expressed as percentages of the total study population.

**Figure 2:**
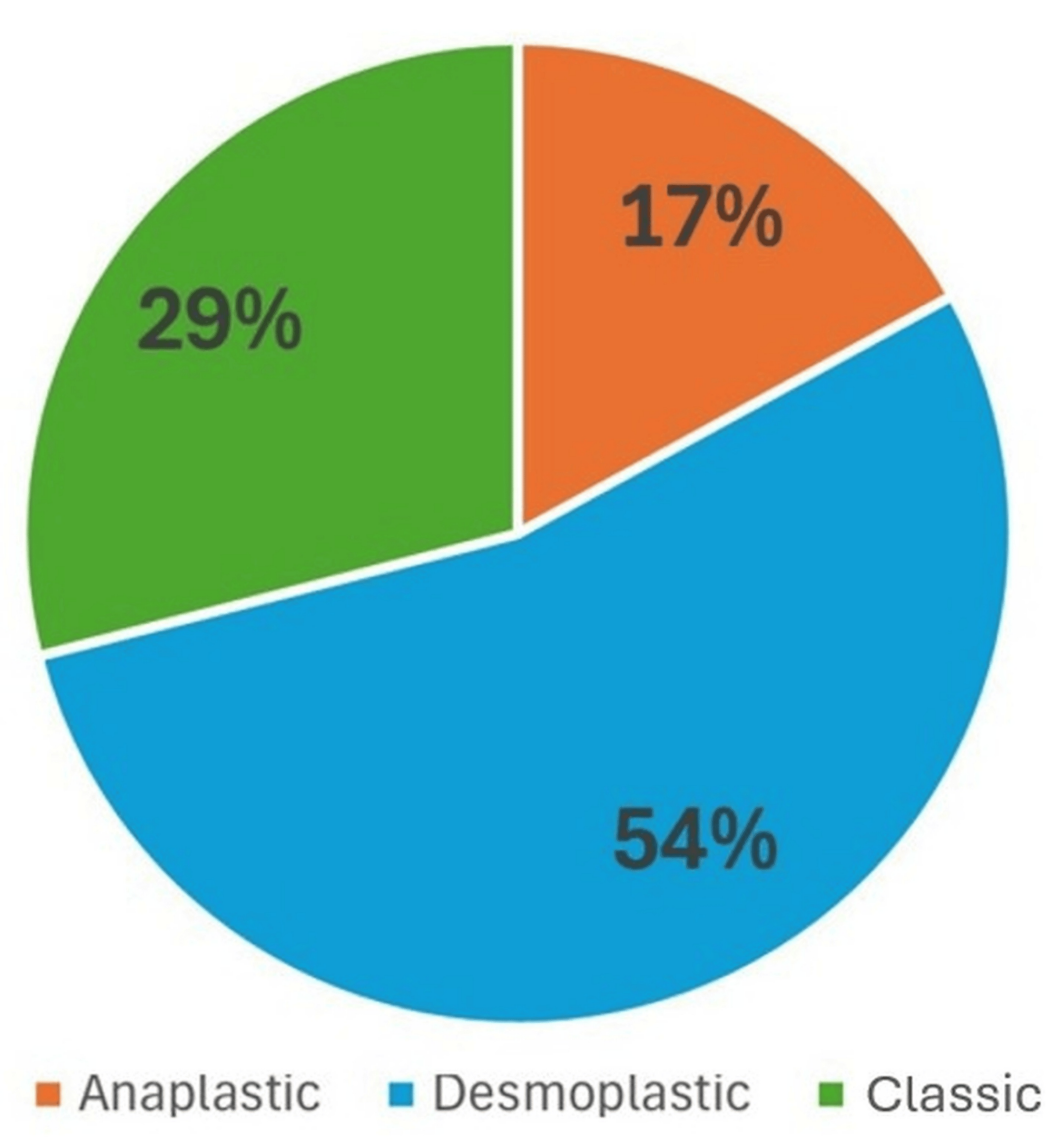
Histological subtype distribution of medulloblastoma. Histopathological subtypes of medulloblastoma in the study population. The desmoplastic subtype was the most frequent (54%), followed by the classic subtype (29%) and the anaplastic subtype (17%). Data are shown as percentages of the total study population.

Tumor localization is shown in Figure 1, while the most common histological variants are shown in Figure 2. As shown in Table 1, homogeneous enhancement on MRI was observed in 15 (62.5%) cases. STR, defined as residual tumor >1.5 cm², was performed in 19 (79.2%) patients, while total resection with residual tumor <1.5 cm² was achieved in only five (20.8%) patients. Chemotherapy was indicated in 23 (95.8%) patients, and 17 (70.8%) received radiotherapy. Three patients (12.5%) were lost to follow-up. Among the remaining 21 patients, 13 (61.9%) died during the observation period.

**Table 1:** Frequency of contrast enhancement on MRI, extent of resection, and administration of chemotherapy and radiotherapy in the study population. Data are presented as absolute frequencies (n) and percentages (%).

Variable	n (%)
Enhancement	
Homogeneous	15 (62.5)
Heterogeneous	3 (12.5)
Minimal	6 (25.0)
Resection grade	
Residual tumor <1.5 cm²	5 (20.8)
Residual tumor >1.5 cm²	19 (79.2)
Chemotherapy	23 (95.8)
Radiotherapy	17 (70.8)

**Figure 3:**
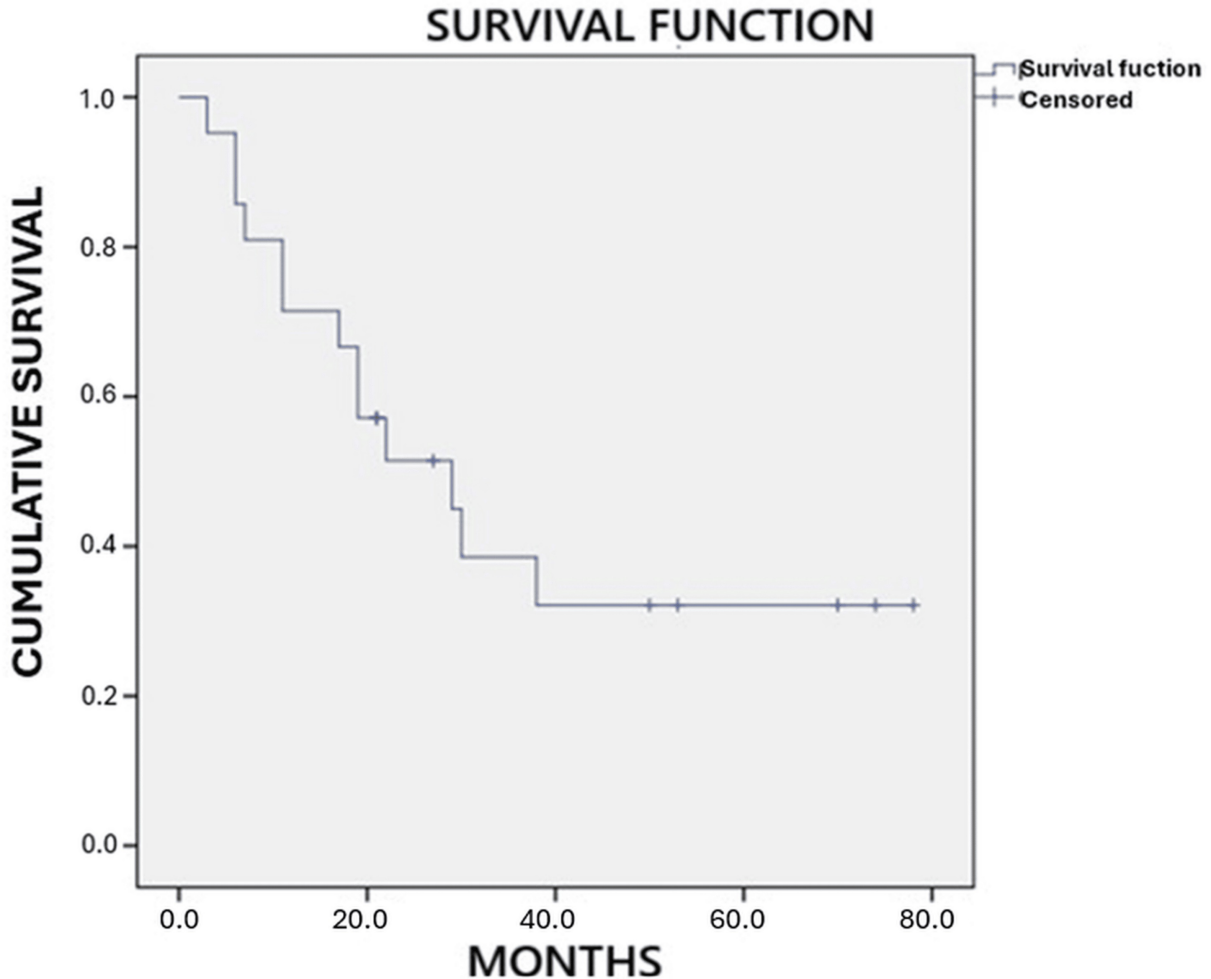
Overall survival curve. Overall survival curve of the pediatric medulloblastoma cohort. The Kaplan-Meier survival function shows the proportion of surviving patients over time, with censoring events indicated by tick marks. Median survival appears to be reached at approximately 30 months, with a plateau observed after 60 months.

The median OS was 32.28 ± 6.8 months (95% CI: 23.9-50.5), with survival rates of 57.2% at 20 months and 38% at 40 months (Figure 3).

Survival

**Figure 4:**
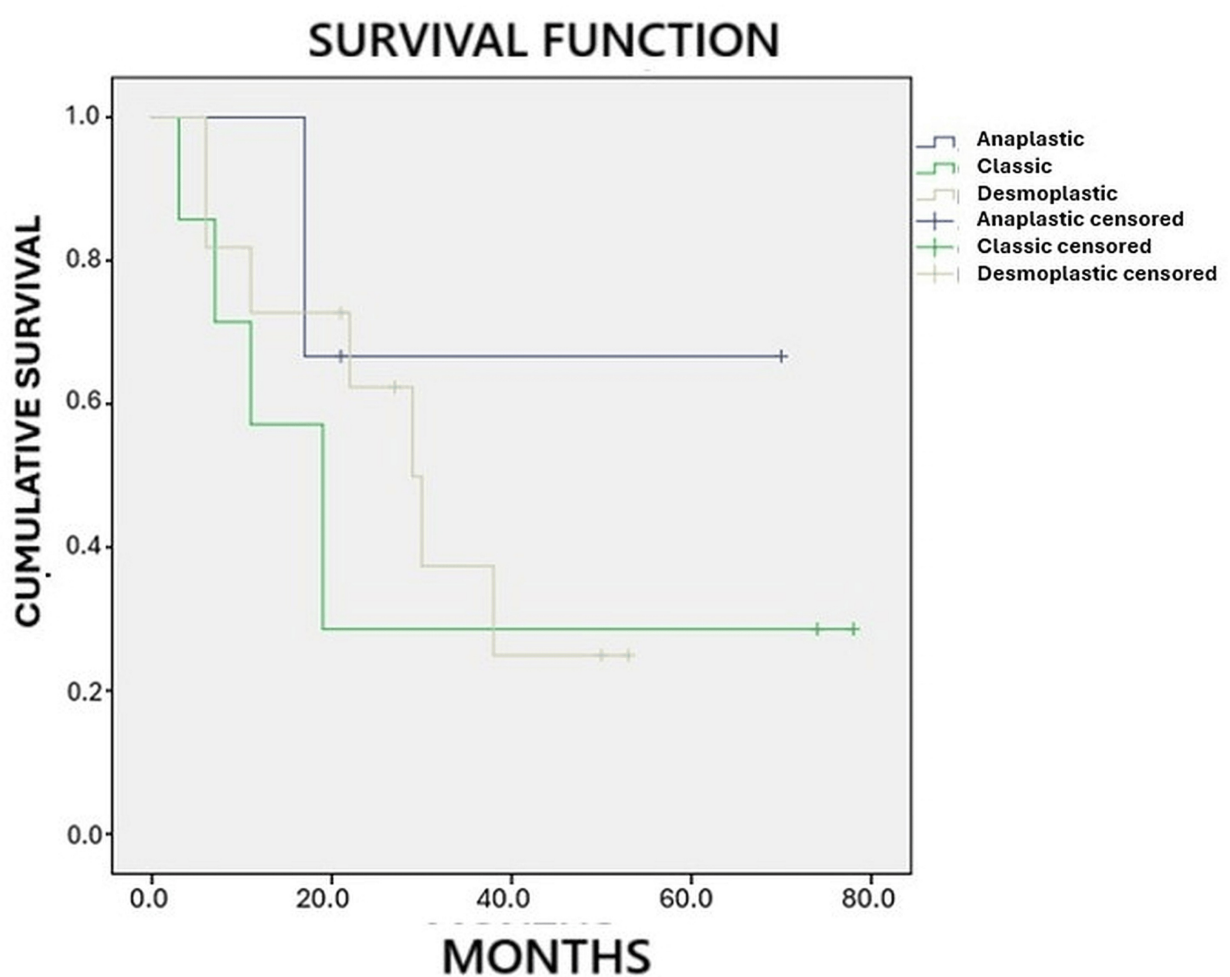
Survival curve according to histological subtype. Kaplan-Meier survival curves by histopathological subtype of medulloblastoma. In this cohort, the anaplastic subtype showed the highest cumulative survival over time, while the classic and desmoplastic subtypes were associated with lower overall survival. Censored data points are indicated by tick marks on the respective curves.

**Figure 5:**
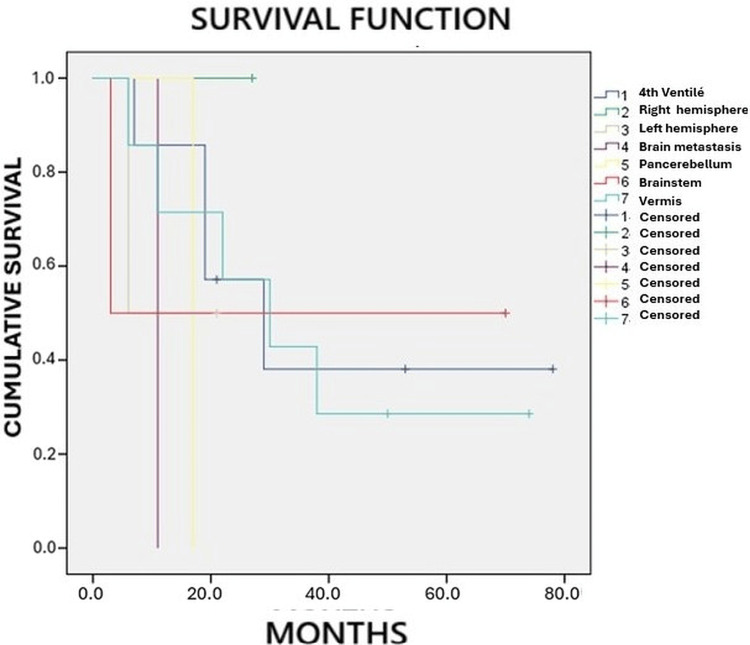
Survival curve according to tumor location. Kaplan-Meier survival curves comparing overall survival according to tumor location in pediatric medulloblastoma. Each curve represents a distinct tumor location, with censored cases indicated by tick marks. The survival trends show notable variability among locations, particularly within the first 30 months of follow-up.

**Figure 6:**
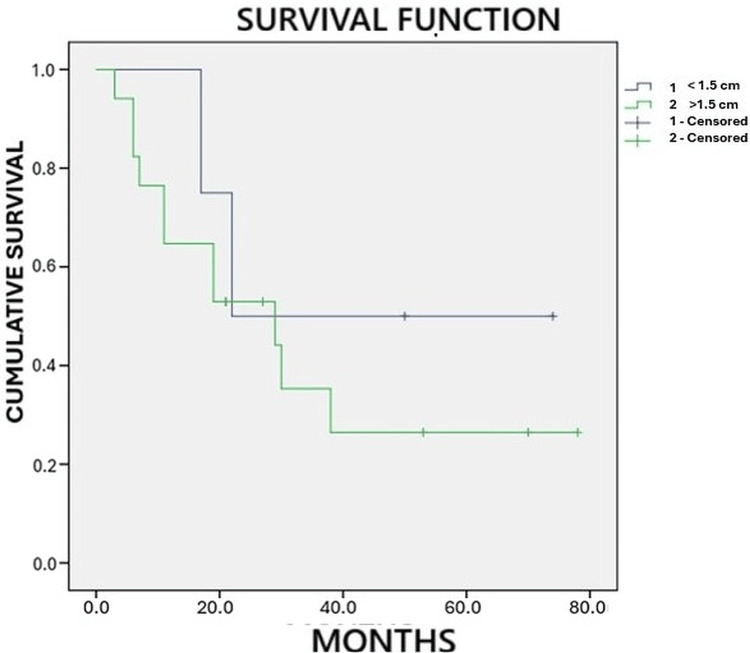
Survival curve according to extent of resection. Kaplan-Meier survival analysis comparing overall survival between two groups of patients with medulloblastoma according to extent of resection. Group 1 demonstrated higher cumulative survival over time compared with Group 2, particularly beyond 20 months of follow-up. Censoring events are marked on the respective survival curves.

Survival by histological subtype is shown in Figure 4. Patients with anaplastic medulloblastoma had a mean survival of 52.33 ± 14.4 months (95% CI: 24.1-80.6), with OS rates of 66.6% at 17 months and 70 months. For the classic subtype, the mean survival was 30.71 ± 11.5 months (95% CI: 8.2-53.2), with OS rates of 57.1% at 12 months and 28.6% at 19 months. Desmoplastic medulloblastoma showed a mean survival of 29.7 ± 19.2 months, with OS rates of 72.7% at 11 months and 45.5% at 38 months. When OS was stratified by tumor location (Figure 5), brainstem tumors had a survival rate of 50% at 3 months, with a mean survival of 36.5 ± 40.38 months. Tumors located in the fourth ventricle showed a survival rate of 42.85% at 19 months, with a mean survival of 30.85 ± 25.82 months. Tumors in the vermis had a survival rate of 42.85% at 30 months, with a mean survival of 33 ± 23.58 months. Figure 6 illustrates OS by extent of resection. Patients who underwent total resection with residual tumor <1.5 cm² had an OS rate of 50% at 22 months and a mean survival of 46.7 ± 13.7 months. In contrast, those who underwent STR with residual tumor >1.5 cm² had an OS rate of 52.9% at 19 months, with a mean survival of 34 ± 7.4 months.

## Discussion

In our study of 24 pediatric patients with medulloblastoma, the majority were male, with a mean age of 7.8 years. The desmoplastic subtype and cerebellar vermis localization were the most frequent findings. STR was performed in most cases, and although chemotherapy and radiotherapy were widely administered, the median OS was 32.28 months. Survival varied by histological subtype, tumor location, and extent of resection.

Interestingly, patients with anaplastic histology demonstrated longer survival in our cohort, which contrasts with most published series in which LCA medulloblastoma is typically associated with a poorer prognosis. Our findings differ from multiple previously published series reporting poorer outcomes in patients with LCA medulloblastoma. Brown HG et al., in a large Pediatric Oncology Group study including 495 medulloblastoma cases, demonstrated significantly worse survival among patients with LCA histology compared with other histological subtypes [13]. Similarly, von Hoff K et al. reported the adverse prognostic impact of LCA variants in pediatric medulloblastoma cohorts [14]. Therefore, the survival pattern observed in our study should be interpreted with caution and may be influenced by the limited sample size, retrospective single-center design, variability in histopathological classification, and lack of molecular subgroup characterization. Additionally, the observed differences did not reach strong statistical significance, limiting the ability to establish definitive prognostic associations.

Medulloblastoma is among the most common malignant tumors of the CNS in the pediatric population, accounting for approximately 20% of all childhood brain tumors. In developed countries, the age-adjusted annual incidence ranges from 0.39 to 0.7 cases per 100,000 children, with regional variability [15]. For example, the incidence in the United States is reported at 0.39 per 100,000, compared with 0.45 per 100,000 in Finland [16]. Most cases are diagnosed in children under 10 years of age, with the highest incidence occurring between 5 and 9 years. The tumor is more prevalent in males, with a male-to-female ratio of approximately 1.7:1 [11].

Historically, medulloblastoma management has relied on multimodal treatment strategies combining surgical resection, radiotherapy, and chemotherapy, which have progressively improved survival outcomes over time [17-19]. Contemporary approaches increasingly incorporate molecular subgrouping and risk stratification to optimize treatment efficacy while minimizing long-term toxicity [19].

Contemporary management of medulloblastoma emphasizes molecular subgrouping, including WNT, SHH, Group 3, and Group 4, and risk stratification to guide personalized treatment strategies that balance therapeutic efficacy with long-term toxicity reduction [20,21]. In addition, modern radiotherapy techniques, such as proton therapy, along with modified chemotherapy regimens and targeted therapies, aim to minimize late complications. In young children, high-dose chemotherapy followed by autologous stem cell rescue is employed to delay or avoid radiotherapy and its associated sequelae [22]. Moreover, advances in surgical techniques have also shifted the focus toward preserving neurological function rather than achieving gross total resection alone, representing a paradigm shift toward more effective and less invasive treatment approaches [23].

Reported survival outcomes across Latin American countries remain heterogeneous, with treatment delays, limited access to radiotherapy, and aggressive histological subtypes contributing to poorer prognosis in several LMIC settings [22-25].

In our study, the sample consisted primarily of males, 16 (66.7%), which aligns with previous reports suggesting a higher incidence of medulloblastoma in males. The mean age of 7.83 years also corresponds with the literature, where this type of tumor is frequently observed in school-age children. Srinivasan VM et al. confirmed this in their study, reporting a mean age of 8 years and a higher frequency in males, with a ratio of 2:1 [1]. Regarding clinical presentation, the most common initial symptom was headache in 10 patients (41.7%), followed by intracranial hypertension in six cases (25%) and ataxia in three (12.5%). These signs and symptoms are consistent with those described by Coluccia D et al., who reported that the most common presenting signs leading to diagnosis include lethargy, vomiting, headache, and truncal or axial ataxia, which often persist for 1.5 to 3 months before diagnosis [3]. This underscores the importance of considering such symptoms during the initial evaluation of children with suspected neurological pathology, as they may be indicative of an underlying brain tumor.

Imaging findings and extent of surgical resection

Homogeneous enhancement on magnetic resonance imaging was observed in 62.5% of patients. The majority, 19 (79.2%), underwent STR with residual tumor greater than 1.5 cm², reflecting the inherent challenges of achieving complete resection, particularly in anatomically critical regions. This limitation highlights the importance of advanced surgical tools, such as neuronavigation, which facilitates precise delineation of tumor boundaries and extent of involvement. In cases where the tumor is adherent to vital structures, the surgical goal is often near-total resection or intentional preservation of residual tumor measuring less than 1.5 cm². Notably, patients who underwent total resection with residual tumor <1.5 cm² exhibited an OS rate of 50% at 22 months, suggesting a potential association between maximal safe resection and improved survival outcomes [26].

Narayan V et al., in a study of 118 patients with a mean age of 12 years, performed total resection in 108 (91.5%) patients and STR in 10 (8.5%) patients. Tumor hemorrhage and heart rate variability were the reasons for STR in these patients. The extent of resection was identified as a very important factor influencing the outcome and prognosis of MB, with the mean OS for the entire group being 82.1 ± 5.7 months [17-18].

Regarding adjuvant treatment, of the 24 patients included, 23 received chemotherapy and 17 (70.8%) underwent radiotherapy. These adjuvant modalities are essential components in the management of medulloblastoma, as multimodal therapy has consistently been associated with improved survival outcomes. The use of radiotherapy, particularly higher doses and focal boosts, has been linked to prolonged survival and reduced recurrence rates. Recognizing the importance of adjuvant therapy is especially critical given its positive impact on disease-free survival. Consequently, emphasis is placed on achieving maximal safe resection, particularly in young children under the age of 3 years, in whom radiotherapy is generally avoided or delayed because of its detrimental effects on neurodevelopment. In this population, treatment strategies typically focus on radiotherapy-sparing or radiotherapy-delaying approaches to preserve long-term neurocognitive function [27]. As a result, chemotherapy-only regimens are generally preferred for infants and children under 3 to 4 years of age [17].

Survival and histological subtype 

The mean OS in our cohort was 32.28 months, with an OS rate of 57.2% at 20 months. Among the 21 patients with follow-up data, 13 (61.9%) died during the observation period. Differences in survival trends were observed according to histological subtype: patients with anaplastic tumors exhibited longer observed mean survival within our cohort (52.33 months), while those with desmoplastic and classic subtypes had mean survivals of 29.7 and 30.71 months, respectively. These findings suggest that histopathological classification may contribute to prognostic assessment; however, molecular biomarkers remain essential for contemporary risk stratification and treatment planning. LCA tumors in infants are frequently associated with high-grade Group 3 molecular features, characterized by poor prognosis and high metastatic potential. In contrast to our cohort findings, DNMB has demonstrated the most favorable survival outcomes in prior studies, whereas the LCA subtype has been associated with the worst prognosis. In a study of 99 cases, Narayan V et al. reported that among 51 patients with classic medulloblastoma, the mean OS was 82.6 ± 7.8 months; among 29 DNMB cases, OS reached 86.7 ± 7.1 months; and among 18 LCA cases, OS was significantly lower at 39.2 ± 6.7 months. One case of MBEN was excluded due to unreported survival data [18].

Survival and tumor location 

Survival outcomes also varied according to tumor location. Brainstem tumors demonstrated a poor prognosis, with a survival rate of 50% at 3 months. In contrast, tumors located in the fourth ventricle and cerebellar vermis were associated with longer mean survival times, underscoring the prognostic relevance of tumor location in clinical progression. In pediatric patients, medulloblastomas typically arise along the midline, particularly in the vermis. Surgical resection is generally more feasible in laterally located lesions, whereas midline tumors involving the brainstem pose greater technical challenges and are associated with lower rates of total resection [19].

Limitations

This study has several limitations that should be considered when interpreting the findings. The retrospective single-center design and limited sample size restricted the ability to perform robust multivariate analyses and may have reduced the statistical power necessary to detect independent prognostic associations. Additionally, loss to follow-up in a subset of patients may have influenced survival estimations and outcome assessment. Molecular subgroup characterization was not available, limiting comparison with contemporary molecularly stratified medulloblastoma series and reducing the precision of biological and prognostic classification. Histopathological evaluation was limited to three subtypes, desmoplastic, classic, and anaplastic, without complementary molecular subgrouping. Given the established prognostic relevance of molecular classification, particularly in pediatric populations in which certain molecular groups may demonstrate more aggressive behavior and increased metastatic potential, the absence of molecular data may obscure the underlying biological heterogeneity of medulloblastoma and limit the accuracy of treatment stratification.

Moreover, potential confounding variables, such as comorbidities, previous treatments, and socioeconomic factors, were not systematically assessed. These variables may significantly influence treatment accessibility, clinical outcomes, and OS, and their omission could have affected the observed associations. Additionally, although this study evaluated clinical, radiological, histopathological, and survival-related characteristics, it did not assess post-treatment quality of life outcomes. Considering the long-term neurological, cognitive, and functional sequelae associated with pediatric medulloblastoma treatment, quality of life assessment should be incorporated into future studies to provide a more comprehensive evaluation of therapeutic outcomes. Therefore, the findings of the present study should be interpreted cautiously and considered primarily exploratory and hypothesis-generating rather than definitive causal prognostic conclusions.

Strengths

Despite these limitations, this study also has several important strengths. Standardized data collection procedures and independent dual-review data extraction were implemented to improve data consistency and reduce information bias. Additionally, the inclusion of consecutive pediatric medulloblastoma cases from a tertiary care center contributes valuable clinical and survival data from a Latin American middle-income setting, a population that remains underrepresented in the neuro-oncology literature. These findings may contribute to improving the characterization of medulloblastoma outcomes in resource-limited settings and support future multicenter investigations.

## Conclusions

The findings of this study underscore the critical importance of a multimodal treatment approach and highlight the need for larger, multicenter studies incorporating long-term follow-up and molecular classification. Comprehensive molecular profiling is essential to refine risk stratification and personalized treatment, thereby optimizing outcomes. Equally important is the need to educate both parents and healthcare providers about the implementation of standardized protocols following the initial diagnosis. This would help minimize delays in referral, ensure timely follow-up, and facilitate prompt initiation of surgical and adjuvant therapies, which are key elements for improving survival rates and preserving quality of life.

The observed high mortality rate, along with significant survival variability based on tumor location and histological subtype, emphasizes the urgency of developing novel therapeutic strategies. Future research should focus on identifying predictive biomarkers to guide treatment response and enhance clinical decision-making in pediatric medulloblastoma.
